# Size-Dependent Properties of Magnetosensitive Polymersomes: Computer Modelling

**DOI:** 10.3390/s19235266

**Published:** 2019-11-29

**Authors:** Aleksandr Ryzhkov, Yuriy Raikher

**Affiliations:** 1 Laboratory of Mechanics of Functional Materials, Institute of Continuous Media Mechanics, Ural Branch, Russian Academy of Sciences, 614068 Perm, Russia; 2 Laboratory of Physics and Mechanics of Soft Matter, Institute of Continuous Media Mechanics, Ural Branch, Russian Academy of Sciences, 614068 Perm, Russia; raikher@icmm.ru

**Keywords:** magnetic polymersomes, magnetic vesicles, magnetic nanoparticles, magnetoactive composites, nanocapsules, coarse-grained molecular dynamics, computer simulation

## Abstract

Magnetosensitive polymersomes, which are amphiphilic polymer capsules whose membranes are filled with magnetic nanoparticles, are prospective objects for drug delivery and manipulations with single cells. A molecular dynamics simulation model that is able to render a detailed account on the structure and shape response of a polymersome to an external magnetic field is used to study a dimensional effect: the dependence of the field-induced deformation on the size of this nanoscale object. It is shown that in the material parameter range that resembles realistic conditions, the strain response of smaller polymersomes, against *a priori* expectations, exceeds that of larger ones. A qualitative explanation for this behavior is proposed.

## 1. Introduction

Synthesis of microscale particles capable of drug transportation and/or active manipulations with cells is one of the most actively developing trends in modern biomedicine [1]. A promising class of such objects, well tunable and controllable, are polymersomes: vesicle-like submicron capsules whose walls are formed by a bilayer membrane built of an amphiphilic block copolymer [2,3,4,5,6,7]. The polymersome might be loaded with biomedical substances (cargo) whereas the membrane might be functionalized to be stimuli-sensitive to a number of factors: pH, temperature, oxidation/reduction, electric/magnetic fields, light, ultrasound, etc. Chemical versatility and stability of polymersomes ensure a variety of their foreseen applications: biosensing systems, nanoreactors, drug carriers with externally activated release, imaging, and even prototypes of artificial cell organelles.

The objects of current study are magnetosensitive polymersomes (magnetopolymersomes, MPSs) whose membrane is modified by introducing inside it magnetic (e.g., maghemite) nanoparticles (MNPs) [8,9,10,11]. Due to the hydrophilic/hydrophobic interplay, the MNPs are confined inside the sandwich structure formed by the inner and outer amphiphilic polymer layers (shells); the width of the intershell gap is adjusted in such a way that it just a bit exceeds the MNP size. With such a construction, magnetodeformational susceptibility of MPSs comes out several orders of magnitude higher than that of any other types of microcapsules.

Nowadays, chemical synthesis of various modifications of MPSs is rapidly developing, and they are available in different shapes and morphologies [12,13]. In [14], membranes of poly(trimethylene carbonate)-*b*-poly(L-glutamic acid) (PTMC-*b*-PGA) with incorporated maghemite ($\gamma -{\mathrm{Fe}}_{2}O_{3}$) nanoparticles are reported. The monolayer, double-layer and multilayer vesicles with poly(styrene)-*b*-poly(acrylic acid) (PS-*b*-PAA) membranes of tuned thickness and stuffed with MNPs are obtained in [15]. The tests demonstrate that the polymersomes with thicker membrane and higher MNP density display enhanced MRI contrast, higher magnetization and better release profile of their drug cargo. In structure investigations, it is found that larger MNPs drift closer to the inner membrane boundary that leads to larger curvature of the latter [16]. Besides dilute systems, preparation of size-controlled assemblies of densely packed submicron PS-*b*-PAA polymersomes is also possible [16].

Now a commonly established fact is that under an applied external field, MPSs notably stretch along the field direction. Moreover, as revealed by small-angle neutron scattering (SANS), the field causes changes in the membrane structure making it anisotropic, and this anisotropy increases with the applied field. The evaluated scattering anisotropy factor is also found to be dependent on the MNP content, size and curvature of the membrane. According to the SANS observations, the field strength at which the field-dependence of the anisotropy factor attains a plateau coincides with that saturating the MNP magnetic moments [14].

The granted biocompatibility of MPSs and experimentally proven opportunity to magnetically control their shape as well as the changes of MNP distribution inside the membrane, commend them as really smart micro-objects for biomedical use. For further progress, one needs a valid concept of complex magnetomechanical and magnetostructural properties of MPSs that, as for now, is virtually nonexistent. A promising, if not the only, way to fill in this gap, i.e., to study and predict the deformational and structure responses of MPSs, is an extensive use of computer simulations. In what follows, we describe a flexible computational model of an MPS and use it to analyze the field-induced shape and volume effects.

## 2. Model and Simulations

A coarse-grained molecular dynamics description of a magnetic polymersome was developed in [17]. Here, we use it to investigate the dependence of magnetodeformational behavior of an MPS on the overall size of the latter.

**Figure 1:**
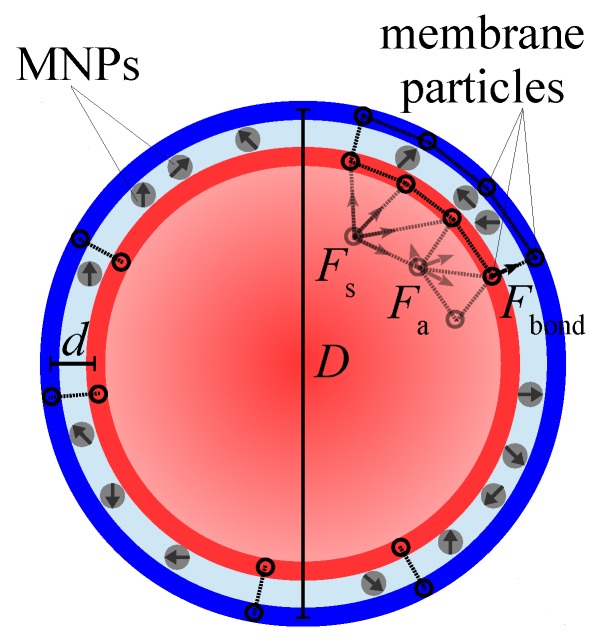
Schematic cross-section of the model MPS. The membrane consists of the internal (red) and external (blue) shells made of equal number of polymer particles (beads) arranged in triangular mesh; the intershell space is filled with MNPs(gray spheres with arrows).

The model MPS is built of the particles of two types: non-magnetic polymer ones (beads) and MNPs. The sandwiched amphiphilic membrane, within which the MNPs are confined, is presented as two nested spherical shells, the inner and outer ones, see Figure 1. Each shell consists of an equal number of beads arranged in a quasi-2D triangular mesh, so that the centers of beads are the mesh nodes; the diameters of the beads are denoted as $d_{\mathrm{in}}$ and $d_{\mathrm{out}}$, respectively, where the subscript indicates the shell.

Inside each shell the bead centers are connected with stiff springs to ensure virtual constancy of the inter-bead distances and, thus, the area of the triangle built of any three neighboring beads. At the surface organized in such a way, any pair of side-abutting triangles is, however, free to fold along their mutual border. In other words, the overall surface area of the shell is conserved but the shell bending elasticity is negligible.

Inside this double-shell membrane, in a number of points uniformly distributed over its surface, the subtending beads (nodes) of the shells are linked with identical linear elastic springs; the stress-free length *h* of the spring defines the equilibrium thickness of the intershell layer. Given that the total membrane thickness is $h+\frac{1}{2}\left(d_{\mathrm{in}}+d_{\mathrm{out}}\right)$, whereas the gap accessible for the MNPs is $h_{\mathrm{gap}}=h-\frac{1}{2}\left(d_{\mathrm{in}}+d_{\mathrm{out}}\right)$. The connectivity parameter $c_{b}$ of the membrane is defined as the ratio of the number of spring-bonded pairs to the whole number of particles in the shell; evidently, diminution of $c_{b}$ enhances the extent of the layer thickness fluctuations.

Inside the MPS membrane, a certain number of monodisperse spherical magnetic nanoparticles is confined, thus making a kind of quasi-2D magnetic fluid. The volume fraction ϕ of the MNPs is defined with respect to the volume of the intershell space where they are allowed to move. From the calculation viewpoint, each MNP is treated as a structureless bead of diameter $d_{p}$ with a built-in magnetic (dipole) moment fixed at its center. The intershell gap $h_{\mathrm{gap}}$ but slightly exceeds $d_{p}$, so that the intershell layer might accommodate only a monolayer of MNPs. In magnetic aspect, the MNPs are coupled with each other as point dipoles; in steric aspect, they interact as soft spheres. The diameters $d_{\mathrm{in}}$ and $d_{\mathrm{out}}$ of the shell beads are chosen is such a way that the shells are impenetrable for the MNPs.

To commence simulation, first, under the above-presented conditions a multi-element object (a model MPS) is constructed as a hollow sphere of outer diameter *D* with a double-shell wall, within which a given number of MNPs is uniformly distributed; the number of the beads in the shells is chosen accordingly. Then all the interparticle interactions are “switched on”, and the model MPS is set in contact with Langevin thermostat that induces Brownian motion of all the elements of the system. Therefore, the thermostat imitates the presence of isothermal solvent (consisting of light-weight molecules) which uniformly fills in all the space outside and inside the MPS.

From this instant the coarse-grained molecular dynamics calculation starts and is carried out until the system comes to equilibrium; the criterion for the latter is stabilization of the overall energy value. This stage yields the basic (field-free) state of the MPS and enables one to obtain its characteristics. Then a uniform magnetic field is imposed, i.e., the Zeeman energy is added to the energy of each magnetic element, and the calculation process is run anew.

Technically, each calculation implies the numerical solution of the set of coupled equations of motion for all the center-of-mass position vectors ${\vec{r}}_{i}\left(t\right)$ where subscript *i* enumerates all the elements of the model MPS, beads or magnetic particles:

(1) $$m_{i}\frac{d^{2}{\vec{r}}_{i}}{dt^{2}}={\vec{F}}_{i}-\zeta \frac{d{\vec{r}}_{i}}{dt}+{\vec{f}}_{i}\left(t\right);$$

here $m_{i}$ is the element mass, ${\vec{F}}_{i}$ the force derived from the total potential energy. The second term in the right-hand side of Equation (1) is the dissipative force with translational friction coefficient ζ, and ${\vec{f}}_{i}\left(t\right)$ is the random force generated by Langevin thermostat.

The force experienced by each bead of any shell is

$${\vec{F}}_{i}={\vec{F}}_{s,\;i}+{\vec{F}}_{a,\;i}+{\vec{F}}_{\mathrm{bond},\;i}+{\vec{F}}_{\mathrm{MNP}-\mathrm{membrane},\;i},$$

with ${\vec{F}}_{s,\;i}$ being the total stretching/compression force on the part of neighboring mesh nodes, ${\vec{F}}_{a,\;i}$ a sum of forces depending on relative change of the areas of the mesh triangles which have the *i*-th node as a common vertex, ${\vec{F}}_{\mathrm{bond},\;i}$ an elastic force entailed by the presence of the bonds connecting the *i*-th node of one shell with the subtending node of another one, ${\vec{F}}_{\mathrm{MNP}-\mathrm{membrane},\;i}$ a total force of soft mutual repulsion between the *i*-th and all the other MNPs, this set of forces is defined by pairwise Weeks-Chandler-Andersen (truncated Lennard-Jones) potential [18].

The force acting on each MNP is

(2) $${\vec{F}}_{i}={\vec{F}}_{\mathrm{dipolar},\;i}+{\vec{F}}_{\mathrm{MNP}-\mathrm{MNP},\;i}+{\vec{F}}_{\mathrm{MNP}-\mathrm{membrane},\;i}.$$

Here ${\vec{F}}_{\mathrm{dipolar},\;i}$ is the sum of pairwise forces derived from the dipolar magnetic interaction of the *i*-th MNP that bears magnetic moment μ directed along unit vector ${\vec{e}}_{i}$ with all the others located at the intercenter distances ${\vec{r}}_{ij}$. This force has the form

(3) $$-\nabla \left[\mu_{0}\mu^{2}\left(\frac{\left({\vec{e}}_{i}\cdot {\vec{e}}_{j}\right)}{r_{ij}^{3}}-\frac{3\left({\vec{e}}_{i}\cdot {\vec{r}}_{ij}\right)\left({\vec{e}}_{j}\cdot {\vec{r}}_{ij}\right)}{r_{ij}^{5}}\right)\right];$$

here $\mu_{0}$ is vacuum permeability. The second and third terms in the right-hand part of Equation (2) are the soft repulsion forces between MNPs and between MNPs and the confining shells, respectively.

Note that for MNPs, besides translational degrees of freedom, the rotational ones matter as well, since the magnetic moment orientations enter the interparticle forces, see Equation (3). The pertinent equations of rotary dynamics with the corresponding torques induced both by the local fields and external field ${\vec{H}}_{0}$ are included in the whole set of equation as well.

All computer simulations were performed with the aid of ESPResSo code [19], the shells were implemented following the scheme proposed in [20]. The computational experiments included preliminary steps to obtain equilibrium distribution of MNPs inside the MPS membrane under zero external field (basic state) and magnetization steps to obtain the changes induced by the applied field. For every MPS size and for every value of the field strength, the calculations were carried out on 10 copies of the model MPS which differed only in initial orientational distribution of the MNP magnetic moments.

The nondimensional parameters which characterize the system under study are as follows. The intensity of the magnetic dipolar interaction

(4) $$\lambda =\mu_{0}\mu^{2}/d_{p}^{3}k_{B}T,$$

is defined as the ratio of energy of magnetic dipolar interaction of two identical MNPs placed at the closest possible distance, i.e., $d_{p}$, to thermal energy with $k_{B}$ being the Boltzmann constant and *T* temperature.

The second parameter is the reference Zeeman energy of an MNP in the field of strength $H_{0}$ scaled with thermal one:

(5) $$\xi =\mu_{0}\mu H_{0}/k_{B}T.$$

Let us clarify the relations between the dimensional parameters of the problem and their nondimensional counterparts used in the calculations. The nanoparticle diameter $d_{p}$ is set to 15 nm that with the typical magnetization of a ferrite (magnetite or maghemite) M=500 emu yields for the MNP magnetic moment $\mu =\left(\pi /6\right)Md_{p}^{3}\approx 8\times 10^{-16}$ emu. Then, assuming room temperature that is the only choice for aqueous solutions, for the dipole interaction parameter (4) one gets λ≈5.

The Langevin argument ξ evaluated for the same conditions comes out is related to dimensional field strength as $\xi \approx 200H_{0}$, so that ξ=10 corresponds to $H_{0}=2$ kOe that is completely feasible value.

The intershell distance *h* is taken as the length unit; in this scale, $d_{p}/h=0.3$ and $h_{\mathrm{gap}}/h=0.35$. With the above-introduced value of the MNP diameter $d_{p}=15$ nm, for the MPS parameters one finds: h≈50 nm and $h_{\mathrm{gap}}\approx 17$ nm. The size of simulated MPSs varies from q≡D/h=6 to q=14 that makes 2.3 time difference; the value of *D* refers to the initial geometry of the MPS, i.e., a sphere. Taking the above-given value of *h*, one finds that the dimensional size of the tested MPS spans from 300 to 700 nm. In the membranes of all the samples, the volume content of MNPs is constant and equal ϕ≈11 vol.%, and the connectivity of the MPS shells is set to $c_{b}=0.2$.

Keeping the MNP size $d_{p}$, dipole parameter λ, layer thickness $h_{\mathrm{gap}}$, volume content ϕ and connectivity $c_{b}$ constant, we ensure that the “quality” of the MPS membranes is the same (at least, before the field is applied). The next section presents the results of simulating the field-induced responses of such MPSs under variation of their overall sizes *D*.

## 3. Results

**Figure 2:**
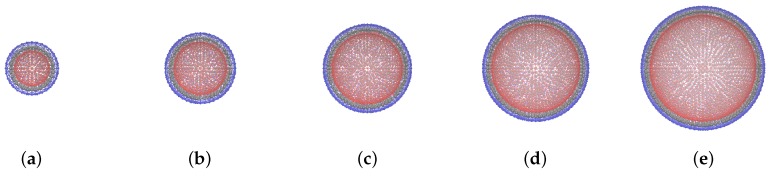
Schematic sketches (drawn at the scale) of model MPSs with nondimensional sizes *q*: 6 (**a**); 8 (**b**); 10 (**c**); 12 (**d**); 14 (**e**); for all samples ϕ≈11% and $c_{b}=0.2$.

The tested MPSs are shown schematically in Figure 2 just for visual comparison.

**Figure 3:**
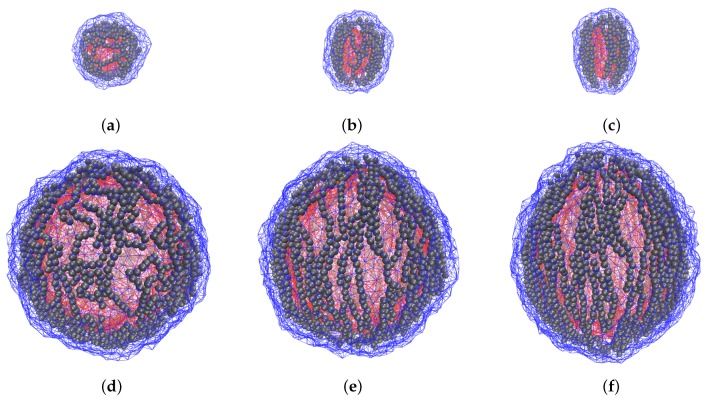
Snapshots of model polymersomes with the size q=6 (**a**)–(**c**) and q=14 (**d**)–(**f**) under applied field ξ=2((**a**) and (**d**)), ξ=6((**b**) and (**e**)), ξ=10 ((**c**) and (**f**)). The field is directed upward; for all samples ϕ≈11% and $c_{b}=0.2$.

A series of snapshots of equilibrium states for MPSs of different sizes under variation of applied field is presented in Figure 3.

**Figure 4:**
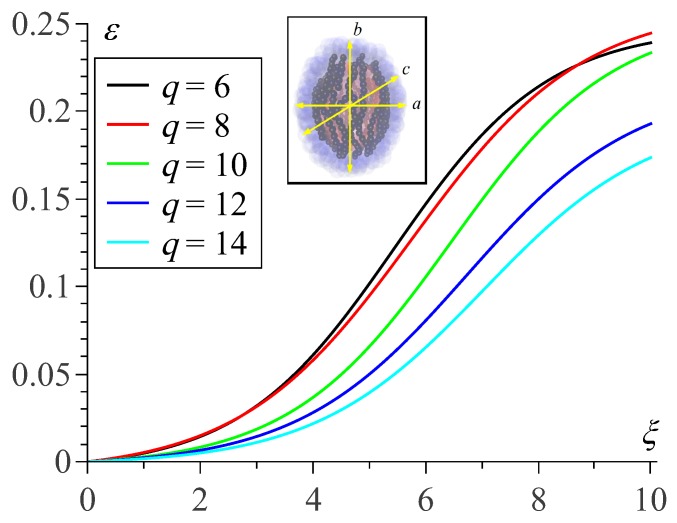
Elongation parameter $\varepsilon =\frac{2b}{\left(a+c\right)}-1$ as a function of external field strength parameter ξ for the MPSs of different initial sizes *q*; all the curves are obtained for ϕ≈11% and $c_{b}=0.2$.

Simulations of the model MPSs under the field point out the common tendency to stretch along the field direction assuming axisymmetric ellipsoidal-like shape. Because of that, it suffices to describe this deformation by a single elongation parameter ε that we define as the ratio of the major semi-axis of the deformed sample to its minor one minus unity. These results are shown in Figure 4.

The presented plots reveal that the smaller polymersome is subjected to magnetization, the greater is its field-induced deformation. Indeed, under field ξ=10 the elongation parameter is ε≈0.25 for an MPS of size q=6 whereas for the largest one (q=14) it is ε≈0.17, i.e., about 30% lower.

**Figure 5:**
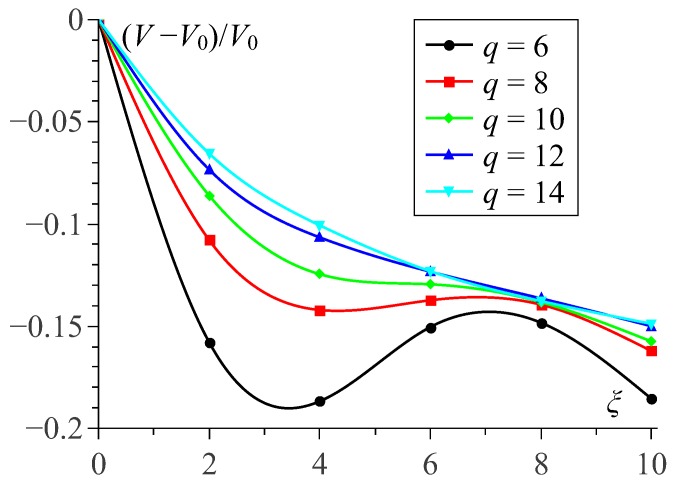
Dependence of the “volume defect” $\left(V-V_{0}\right)/V_{0}$ of the MPS cavity on applied magnetic field ξ; for all samples ϕ≈11% and $c_{b}=0.2$.

Being soft hollow objects, MPSs do not conserve neither their overall nor inner volume under deformation. In view of the drug delivery application, it is interesting to consider the field-induced volume changes of the MPS. The estimation is obtained from simulations by taking any actual configuration of the polymersome and building up a nested polyhedron whose vertexes are fixed at the the centers of the beads of the inner shell. These results are shown in Figure 5, where $V_{0}$ corresponds to the basic state of the MPS and is approximately equal to the volume of a sphere of diameter D−2h. As seen, small polymersomes demonstrate a non-monotonic dependence of the “volume defect” $\left(V\left(\xi \right)-V_{0}\right)/V_{0}$ on the field strength whereas larger ones show regular decreasing behavior.

## 4. Discussion

The coarse-grained molecular dynamics model is applied to investigate the field-induced overall deformation of an MPS and the internal restructuring accompanying it. This enables one to understand the dimension effect: the influence of the size factor on the polymersomes with the same properties of their membranes. The obtained results point out that in these objects the overall changes (shape anisotropy, volume defect) are essentially determined by the structure rearrangements which take place in the magnetically active membrane. As the snapshots of Figure 3 show, the MNPs, formerly dwelling in randomly oriented loose aggregates, re-group and unite in well-formed chains fairly well aligned with the direction of the imposed field; the increase of the field enhances the straightness of the chains. Therefore, one comes to a conclusion that the field-induced chaining of the particles is the main mechanism of MPS elongation. Moreover, it is clear that the equilibrium shape of an MPS is established as a result of interplay between the magnetostatic forces which group the particles and align the chains, and the steric forces which maintain the MNPs confinement in the intra-membrane space.

At a first sight, in the membrane of a larger polymersome (lower curvature), the nanoparticles are more free to align, and, thus, in such an object the conditions for the field-induced elongation are more favorable. However, as the simulation results presented in Figure 4 show, our modelling points out the opposite tendency. To explain this, in below we present qualitative considerations which, if not being exhaustive, should be an important part of the effect.

In our view, the found dependence is a manifestation of the anisotropic character of the dipole-dipole interaction in a confined geometry of a spherical layer. As known, the dipolar particles with parallel orientation of their magnetic moments attract each other if positioned in a “head-to-tail” pattern and experience mutual repulsion in “side-by-side” configuration. More broadly, the particles repel each other if their center-to-center vector is inclined to the field under the angles $\Delta \psi \approx {\left(90\pm 35\right)}^{\circ }$ and are mutually attracted in all the other positions.

When such particles are enclosed in a spherical layer, which is the case of an MPS subjected to a strong field, the situations in the “equatorial” and “polar” zones of the membrane are qualitatively different. (For convenience, we liken an MPS to a globe whose poles are at the points where the direction of external field is parallel to the surface normal.) Indeed, as long as the MPS is sufficiently large $D\gg d_{p}$, those MNPs that inhabit the equatorial zone, are in quite favorable situation for building meridional chains of the “head-to-tail” structure. Meanwhile, for those which at zero field occupied the near-polar zone, the situation strongly depends on the curvature of the layer.

**Figure 6:**
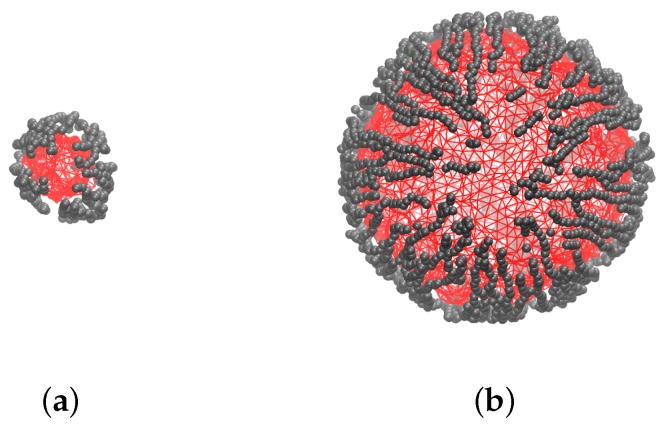
Top views of the MPSs with sizes q=6 (**a**) and 14 (**b**) magnetized by field ξ=10; to improve vision, the outer shell is not shown; these top views correspond to the side views given in Figure 3c,f.

At high curvature (small MPS), virtually all feasible center-to-center vectors fall outside the Δψ interval. This implies that the formerly “polar” particles are attracted by the ends of the meridional chains, drift in their directions, and, thus, deplete the polar zone. At low curvature (large MPS), for a notable number of particles in the near-polar zone have their center-to-center vectors inside the Δψ interval and, thus, experience mutual repulsion. Instead of joining long meridional chains, those MNPs would be repelled by their ends and would stably dwell in the polar zone as single entities or very short weakly linked aggregates. An illustration for this conclusion is given by snapshots in Figure 6.

Evidently, those non-chained particles do not contribute to the stretching effect and by that reduce to some extent the number and/or length of the formed chains. Moreover, the repulsion exerted by those “polar” particles on the nearly-situated chains should extend the MPS in the direction transverse to the field direction and, albeit weakly, but also contribute to diminution of the field-induced stretching of larger MPSs. In Appendix A we propose a “toy” model that justifies the effect of this chain-length reduction on the size-dependent magnetic deformation of MPSs.

Another effect that is worth of discussion is the non-monotonic field dependence of the inner volume of small polymersomes, see Figure 5. In general, as it is could be shown analytically, when a sphere is deformed to spheroid under condition of constancy of its lateral area, the “volume defect” is always negative. Therefore, the found negative volume change, see Figure 5, confirms the assumption of high surface tension of the membrane shells. Looking to details, we surmise that the initial stretching of the MPS in the low field range (ξ=0 to ≈3), see Figure 3 (**a**), where the chains are very curvilinear, is due to orientation of some chain fragments without breaking their overall structure arrangement.

In small polymersomes, on further growth of the field (ξ≈4 to ≈8), the structure rearrangement requires numerous breaks of the formerly existing chains. When the extensive chain rupture takes place, the particle structure becomes more loose, and this transformation, although not completely (see curve for q=6 in Figure 5), but to some extent reduces the volume defect. As soon as the chain fragments unite in a new conformations which are plausible for making quasi-meridional lines, the diminution of *V* resumes. Finally, the field-induced restructuring results in building up inside the membrane a barrel-like superstructure (we remind of the afore-mentioned effect of depleting the polar zones) that makes a small MPS to efficiently stretch along the field direction, see Figure 3c. In large polymersomes, the already existing chains have much more freedom for their conformational changes and perform them with almost no breaks. Therefore the initially formed aggregates gradually reorganize in long-chain patterns; because of that, in such an MPS compression of the inner cavity goes in monotonic way, see plots for q=12 and q=14 in Figure 5 and Figure 3d–f.

## 5. Conclusions

The size effect, i.e., the dependence of structure and deformation of a magnetic polymersome on the applied field strength is studied with the aid of a coarse-grained model. It is shown that in the realistic size range, the field-induced strain—the polymersomes elongation along the applied field—is the higher the smaller the objects. This tendency is related to the arrangement and size of the nanoparticle chains which form and align in the polymersome membrane under magnetization. The field-induced change of the polymersome inner cavity volume is negative, and for small polymersomes its field dependence turns out to be non-monotonic.

The presented results infer that in terms of specific effects—the field-induced strain as well as the volume defect—smaller polymersomes turn out to be more efficient than the larger ones. From application view point, the volume defect is important, in the first place, for drug delivery. The smallness of the obtained value indicates that polymersomes of the considered kind hardly have wide prospects. On the other hand, the 20–25% strain effect might fit in a fairly good way the requirements for cell membrane stimulations which are realized either via turning an already stretched polymersome by a rotating field or by inducing its periodic elongation in the given direction with the aid of alternating field. Certainly, the obtained results on deformational properties of magnetic polymersomes, apart from static cases, might also provide reliable estimates for sufficiently low-frequency magnetodynamic regimes.

Besides the above-presented particular points, it is worth noting that the calculation model that we use, may be easily modified and/or extended in many ways that is an advantage if one would need to describe a magnetic polymersome in more detail.

## Figures and Tables

**Figure A1:**
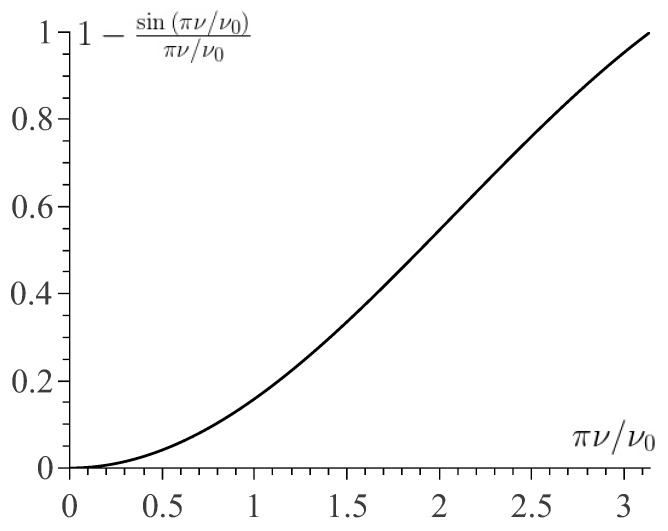
The factor depending on the relative chain length in Equation (A4).

## Abbreviations

The following abbreviations are used in this manuscript:

MNP	magnetic nanoparticle
MPS	magnetic polymersome
MRI	magnetic resonance imaging
PS-*b*-PAA	poly(styrene)-*b*-poly(acrylic acid)
PTMC-*b*-PGA	poly(trimethylene carbonate)-*b*-poly(L-glutamic acid)
SANS	small angle neutron scattering

## Appendix A

Consider an assembly of identical single-domain spherical particles coupled with each other via a sufficiently strong dipolar potential. Under an imposed external field, the basic state (lowest energy) configuration of such a system is a perfectly straight line parallel to the field direction. Evidently, any distortion of this contour enhances the energy of the chain and generates the forces striving to recover the basic state. If the chain is confined, these forces are exerted on the boundaries/walls which restrict its conformational freedom. This is the case of a magnetized MPS: in a strong external field the MNPs align their magnetic moments with the field and aggregate in chains but, being inside the membrane, those chains come out arc-shaped with the curvature equal to that of the membrane in the “meridional” plane, i.e., the plane that contains the field vector and passes through the MPS center. The tendency of the confined chains to straighten, imparts to the membrane an excess of surface energy, and this energy diminishes as the MPS stretches in the field direction.

In an MPS, the approximate volume of spherical layer accessible for MNPs is $V=\pi D^{2}h_{\mathrm{gap}}$, whereas the total volume of all *N* particles is $\left(\pi /6\right)d_{p}^{3}N$. Taking into account that the intershell gap is close to the MNP diameter ($h_{\mathrm{gap}}\approx d_{p}$), the volume fraction of MNPs comes out as $\phi =Nd_{p}^{2}/6D^{2}$. We remind that the above-presented modelling is performed under condition ϕ=const.

Let us assume that the chains stretch meridionally and they are symmetrical with respect to equator of the membrane, so that the chain length is $\nu d_{p}$; the maximal value of the length is $\nu_{0}=\pi D/2d_{p}$, i.e., the chain makes a pole-to-pole semi-circle. The expression for energy ΔU for a model chain of constant curvature could be obtained from general expressions for long dipolar chains, see [21], for example, and taken in the form

(A1) $$\Delta U\approx 6\zeta \left(3\right)\left(\nu \mu^{2}/d_{p}^{3}\right)\left[1-\cos^{2}\vartheta \right].$$

As the chain is considered to be long enough (say, ν>10), it is described in continual limit; here ζ(3) is Riemann’s function and ϑ the local angle between the tangent to the contour of the chain and the field direction; note that we count ΔU from the lowest energy, where $\cos^{2}\vartheta =1$. Under assumption of constant curvature of the arc (2/D), the cosine function in expression (A1) could be averaged over the chain and yields

(A2) $$\cos^{2}\vartheta =\frac{1}{2}\left[1+\frac{\sin\left(\pi \nu /\nu_{0}\right)}{\pi \nu /\nu_{0}}\right],$$

so that substitution to (A1) gives

(A3) $$\Delta U\approx 3\zeta \left(3\right)\frac{\nu \mu^{2}}{d_{p}^{3}}\left[1-\frac{\sin\left(\pi \nu /\nu_{0}\right)}{\pi \nu /\nu_{0}}\right].$$

The part of the membrane surface per one chain is $\Delta S=\pi D^{2}\nu /N=\pi \nu d_{p}^{2}/24\phi$. Then the surface energy density in question is

(A4) $$\sigma =\Delta U/\Delta S=72\zeta \left(3\right)\frac{\phi \mu^{2}}{d_{p}^{5}}\left[1-\frac{\sin\left(\pi \nu /\nu_{0}\right)}{\pi \nu /\nu_{0}}\right].$$

As seen, in expression (A4) the coefficient before the bracket is independent of the MPS structure. Had the ratio $\nu /\nu_{0}$ been the same for any of the considered MPSs, then this independence would have been inherent to the bracket as well. However, as pointed out in Section 4, the larger the MPS diameter *D*, the greater number of MNPs does not take part in stretching. This means that in larger MPSs the ratio $\nu /\nu_{0}$ is lower than in smaller ones. This entails diminution of the value of the factor in brackets and, thus, the decrease of the excess of the magnetic surface energy σ that, in our view, is the driving mechanism of the field-induced stretching of MPSs. To illustrate this, the ν-dependent function of Equation (A4) is presented in Figure A1.
